# Carbon starvation of *Pseudomonas aeruginosa* biofilms selects for dispersal insensitive mutants

**DOI:** 10.1186/s12866-021-02318-8

**Published:** 2021-09-22

**Authors:** Harikrishnan A. S. Nair, Sujatha Subramoni, Wee Han Poh, Nabilah Taqiah Binte Hasnuddin, Martin Tay, Michael Givskov, Tim Tolker-Nielsen, Staffan Kjelleberg, Diane McDougald, Scott A. Rice

**Affiliations:** 1 The Singapore Centre for Environmental Life Sciences Engineering, Singapore, Singapore; 2 Interdisciplinary Graduate School, Singapore, Singapore; 3 Present address: Eppendorf AG, Barkhausenweg 1, 22339 Hamburg, Germany; 4 Present address: Public Utilities Board, Government of Singapore, Singapore, Singapore; 5 Costerton Biofilm Center, Faculty of Health and Medical Sciences, University of Copenhagen, Copenhagen, Denmark; 6 School of Biological Sciences, Nanyang Technological University, Singapore, Singapore; 7 The Ithree Institute, University of Technology Sydney, Sydney, Australia

**Keywords:** Biofilm development, C-di-GMP, Bioreporter, *Pseudomonas aeruginosa*, Image-based quantification, Starvation, Morphotypic variants, Dispersal

## Abstract

**Background:**

Biofilms disperse in response to specific environmental cues, such as reduced oxygen concentration, changes in nutrient concentration and exposure to nitric oxide. Interestingly, biofilms do not completely disperse under these conditions, which is generally attributed to physiological heterogeneity of the biofilm. However, our results suggest that genetic heterogeneity also plays an important role in the non-dispersing population of *P. aeruginosa* in biofilms after nutrient starvation.

**Results:**

In this study, 12.2% of the biofilm failed to disperse after 4 d of continuous starvation-induced dispersal. Cells were recovered from the dispersal phase as well as the remaining biofilm. For 96 h starved biofilms, rugose small colony variants (RSCV) were found to be present in the biofilm, but were not observed in the dispersal effluent. In contrast, wild type and small colony variants (SCV) were found in high numbers in the dispersal phase. Genome sequencing of these variants showed that most had single nucleotide mutations in genes associated with biofilm formation, e.g. in *wspF, pilT*, *fha1* and *aguR*. Complementation of those mutations restored starvation-induced dispersal from the biofilms. Because c-di-GMP is linked to biofilm formation and dispersal, we introduced a c-di-GMP reporter into the wild-type *P. aeruginosa* and monitored green fluorescent protein (GFP) expression before and after starvation-induced dispersal. Post dispersal, the microcolonies were smaller and significantly brighter in GFP intensity, suggesting the relative concentration of c-di-GMP per cell within the microcolonies was also increased. Furthermore, only the RSCV showed increased c-di-GMP, while wild type and SCV were no different from the parental strain.

**Conclusions:**

This suggests that while starvation can induce dispersal from the biofilm, it also results in strong selection for mutants that overproduce c-di-GMP and that fail to disperse in response to the dispersal cue, starvation.

## Background

Upon maturation, biofilm microcolonies release cells in an active process called dispersal, which results in dispersal cells colonising new niches [1]. Bacteria typically disperse from biofilms in response to environmental cues, such as nutrient starvation, oxygen limitation and some signal molecules, e.g. nitric oxide (NO) [2–4]. Biofilm dispersal is an active, energy requiring process that is regulated by multiple molecular pathways [5, 6]. Studies of biofilm dispersal are of broad interest, especially where manipulation of the dispersal response can be exploited to control biofilms in clinical and industrial settings. For example, NO releasing hydrophobic polymers are known to reduce adhesion of bacteria to indwelling medical devices such as catheters and arteriovenous grafts [7]. While it is clear that these cues or pathways can be manipulated for biofilm control, e.g. by exposing biofilms to exogenously added NO donors, exposure to dispersing conditions rarely results in complete biofilm dispersal [8, 9]. Fluctuations in nutrient concentrations (increases or decreases), also trigger biofilm dispersal, although, as observed for NO, dispersal is not complete and some biofilm remains [3, 10, 11]. A monospecies biofilm growing on a single carbon substrate will develop zones of differing oxygen or nutrient availability, e.g. oxygenic, limited oxygenic and anoxic zones, due to incomplete diffusion of oxygen from the medium or as a consequence of rapid utilisation by the bacteria within the biofilm [12–14]. Thus, a facultative aerobic bacterial biofilm may grow aerobically in the oxygenic zone and by fermentation or oxygen-independent respiration in the anoxic zone [12, 15]. Hence, dispersal as a consequence of these environmental cues reflects cellular physiology and it is likely that incomplete dispersal is a reflection of physiological heterogeneity across the biofilm [16].

We have previously shown that biofilms of *P. aeruginosa* disperse upon carbon source starvation and that starvation results in a rapid reduction in c-di-GMP [4]. We further showed that dispersal induced as a result of carbon source starvation was an active, energy requiring process [10]. While carbon source limitation resulted in dispersal, approximately 40% of the biofilm biomass remained, even after 24 h of starvation. It is also frequently observed that mature biofilms produce morphotypic variants, in some cases up to 60% of the recovered isolates and that the appearance of these variants is associated with the dispersal phase of biofilm development [17–21]. Such morphotypic variants are often better at forming biofilms than the parental strain and such variants can outcompete the parental strains when co-cultured [22]. Here, we investigated biofilms of *P. aeruginosa* after starvation-induced dispersal and show that the non-dispersing biomass has a high proportion of morphotypic variants and that these variants have SNPs in genes associated with biofilm formation, in particular, genes associated with the production or degradation of c-di-GMP.

## Results

### Biofilm dispersal due to starvation is incomplete

**Fig. 1:**
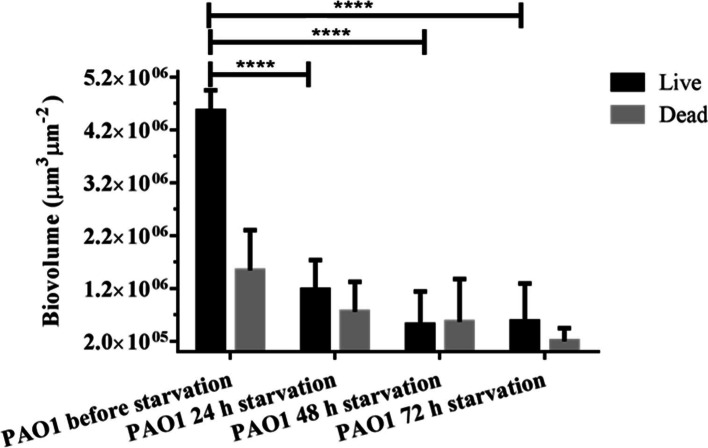
Characterisation of *P. aeruginosa* biofilms during starvation. The biofilm was stained for viability with the BacLight™ Live-Dead® reagents, and the biovolume was calculated as average of 3 independent experiments from CLSM images using the Imaris software package. For each experiment 15 CLSM images were taken. Error bars represent standard deviations (*n* = 3). Asterisks indicate values were significantly different between samples (Two-way ANOVA followed by Sidak’s post-test; **** < P 0.0001)

*P. aeruginosa* biofilms were formed in flow cells for 4 d, after which time starvation was induced by replacing the medium with glucose-free M9. The biovolume was calculated using image-based quantification after staining for live and dead cell populations. Biovolumes of live cells decreased from 4.55 × 10^6^ to 1.18 × 10^6^ μm^3^ μm^− 2^ within 24 h of the start of starvation, indicating significant dispersal in response to starvation (Fig. 1). The biomass of dead cells decreased from 1.4 × 10^6^ to 2.6 × 10^5^ μm^3^ μm^− 2^ after 24 h of starvation. However, after 48 h and 72 h of starvation, the remaining live cell biofilm biovolume was approximately 5.13 × 10^5^ μm^3^ μm^− 2^ and 5.57 × 10^5^ μm^3^ μm^− 2^, respectively. The biomass of dead cells was reduced to 2 × 10^5^ at the end of 72 h of starvation. Thus, although there was substantial dispersal upon starvation, much of the biomass (12.2%) remained attached in the flow cell (Fig. 1). In contrast, we have shown previously that biovolume per unit area of *P. aeruginosa* biofilms increases steadily until day 4 and remains constant until day 7 in the absence of starvation [23]. Therefore, for biofilms under natural nutrient conditions there is no appreciable dispersal until day 7.

### Characterisation of morphotypic variants isolated from starved biofilms

**Fig. 2:**
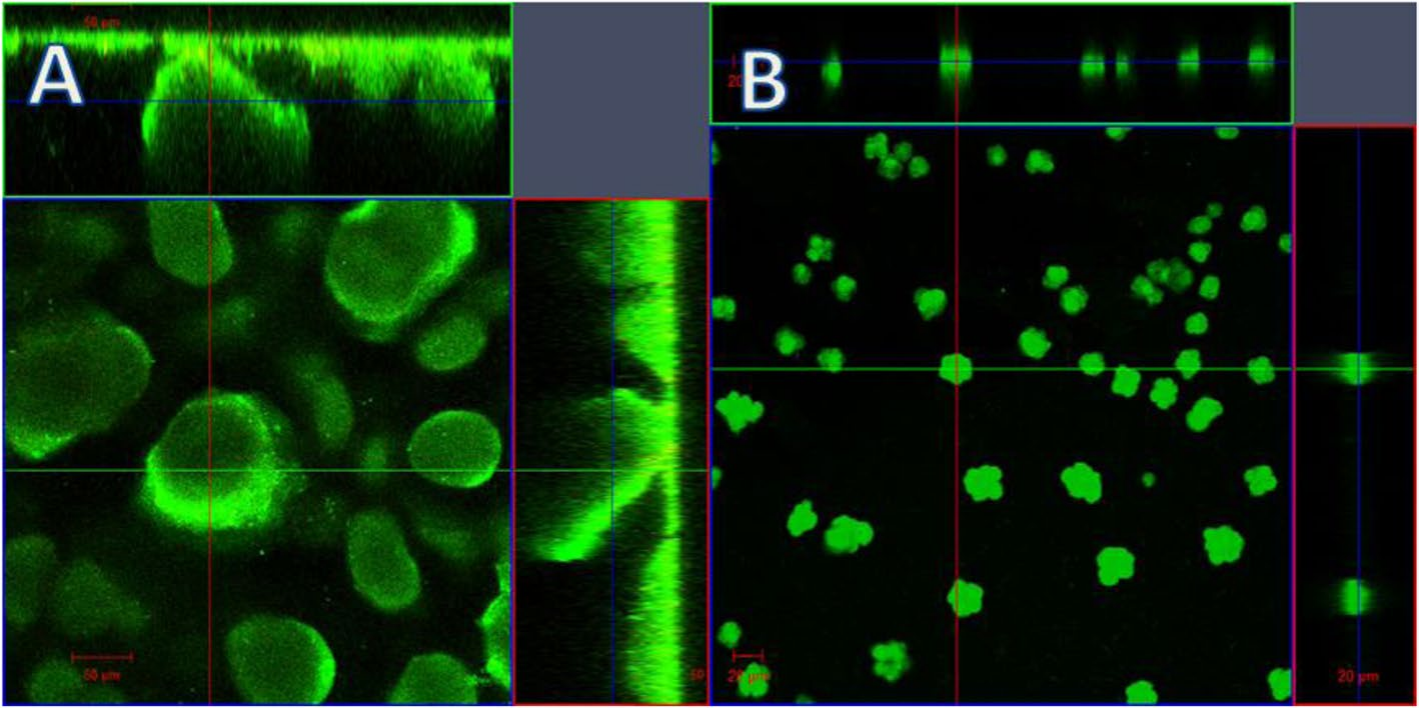
Orthogonal view of *P. aeruginosa* microcolonies before and after starvation. A) biofilm before starvation B) colonies after 72 h of starvation. *P. aeruginosa* contained the c-di-GMP responsive reporter construct as described [39]. Orthogonal view showing top panel *x-z* plane, right *y-z* and middle corresponding *x-y* plane. Magnification 20x. Scalebar: 50 μm (A) or 20 μm (B)

When biofilms containing the pCdrA*::gfp* (ASV) reporter were imaged after 24 h of starvation, it was observed that the remaining biomass was strongly fluorescent, suggesting that those microcolonies had very high intracellular c-di-GMP levels (Fig. 2). It has been previously shown that biofilm dispersal is associated with the formation of morphotypic variants [18, 24] and such variants often are mutants that overproduce c-di-GMP [25, 26].

**Fig. 3:**
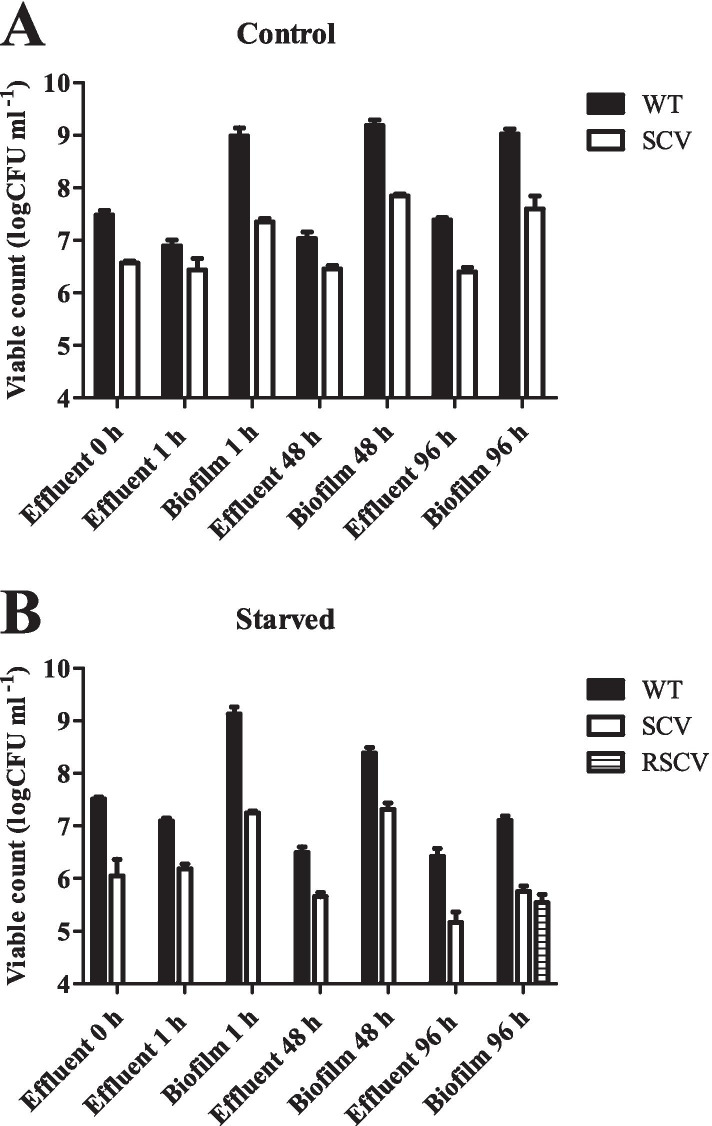
Colony variants of *P. aeruginosa* during biofilm starvation. Effluent from biofilms pre- and post- starvation was serially diluted and plated onto LB agar for enumeration. For each time point, the biomass was also harvested from the flow cells, serially diluted and plated onto LB agar to ennumerate morphotypic variants. Independent flow-cells were run for each time point for control (A) and starved (B) conditions

The biofilm cells formed three distinct morphotypes, one that was similar to the WT *P. aeruginosa*, small colony variants (SCVs) and rugose small colony variants (RSCVs). Therefore, biofilms were collected after 4 d of starvation and cells were serially diluted and plated onto agar plates. The cell numbers were stable at 48 h and 72 h in our earlier experiment; therefore we wanted to check a longer time point and hence chose 96 h over 72 h for enumeration. Prior to starvation, the effluent had high numbers of WT *P. aeruginosa* (3.2 × 10^7^ cfu ml^− 1^) (Fig. 3) and a small number (1.9 × 10^6^ cfu ml^− 1^) of SCVs. After 1 h of starvation, the effluent contained 1.2 × 10^7^ cfu ml^− 1^ wild type and 1.6 × 10^6^ cfu ml^− 1^ SCV. A similar trend was observed after 24 h, 48 h and 96 h of starvation, where the effluent always contained a higher number of WT *P. aeruginosa* and relatively fewer SCVs. For each time-point separate flow cells were cultivated, the flow cell was disconnected and flushed with medium to extract biomass. The biofilm biomass obtained after 1 h, 24 h, 48 h and 96 h of starvation showed a similar trend of higher levels of WT *P. aeruginosa* and lower levels of SCVs. Only the biofilm biomass extracted after 96 h starvation was found to contain RSCVs. At this time point the numbers of bacteria in the extracted biomass included 4 × 10^5^ cfu ml^− 1^ RSCVs, 6 × 10^5^ cfu ml^− 1^ SCVs and 1.3 × 10^7^ cfu ml^− 1^ WT bacteria. The lower numbers of WT bacteria enumerated at 96 h enabled detection of RSCVs in the biofilm biomass at this timepoint. Control biofilms without starvation were also tested and the number of variants were enumerated from both effluent and biofilm biomass for comparison as shown (Fig. 3).

Five representatives of each morphotype were genome sequenced. The WT isolates (WT1–5) harboured one or more mutations (Table 1). For example, the WT1 had a deletion in *fliH* and a substitution in *mexT*. WT2 had only one mutation, an insertional mutation in PA4321, a hypothetical gene. WT3 and WT5 had several SNPs in *pilT*, which is known to be important for twitching motility [27, 28]. WT4 had an insertional mutation in *fha1* coding for a component of the type VI secretion system [29]. SCV1 and SCV2 had mutations in *wspF*, coding for a methylesterase protein of the chemosensory signal-transduction system [30, 31]. Loss of WspF function leads to over expression of the diguanylate cyclase WspR and hence, increased biofilm formation [30]. Some of these morphotypic variants contained additional mutations, including *fha1* in SCV1 and SCV3 and *flhA*, involved in flagella biosynthesis in SCV3, SCV4 and SCV5 [32]. All of the RSCVs contained a mutation in *wspF* and this mutation was consistent with their rough colony morphotype as described previously [22, 33]. Additional mutations in the RSCVs include *aguR* in RSCV2, mutation of PA4321 along with *wspF* in RSCV3 and mutation of PA4543 in RSCV4. RSCV5 and RSCV1 were genotypically the same, containing a single nucleotide variant (SNV) at the same position in the *wspF* gene.

**Table 1:**
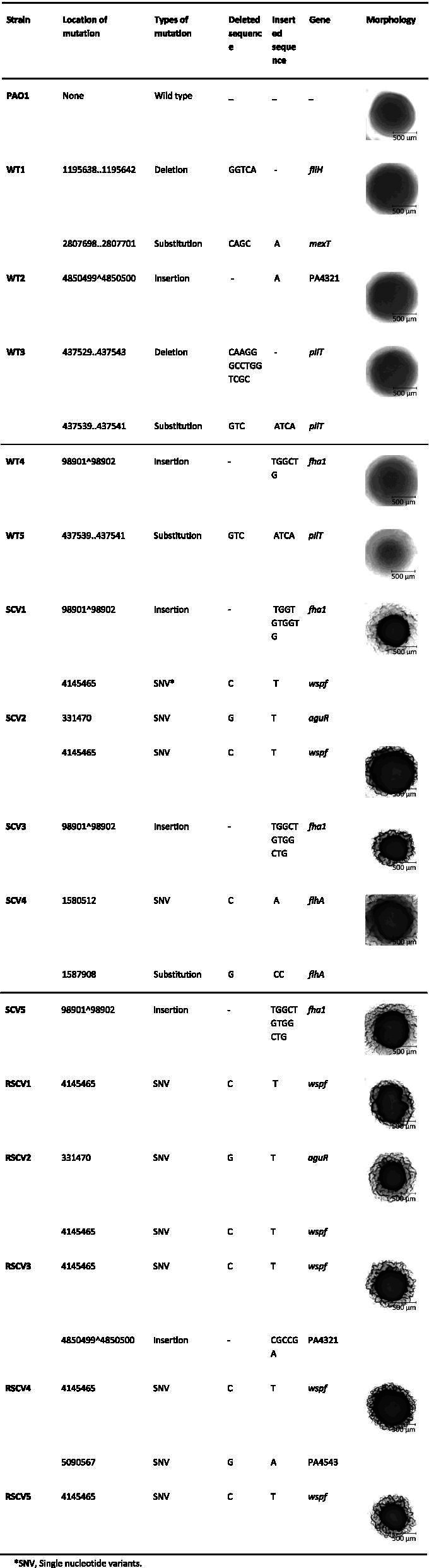
Identification of genetic differences between the *P. aeruginosa* wild type and non-dispersing isolates

**Fig. 4:**
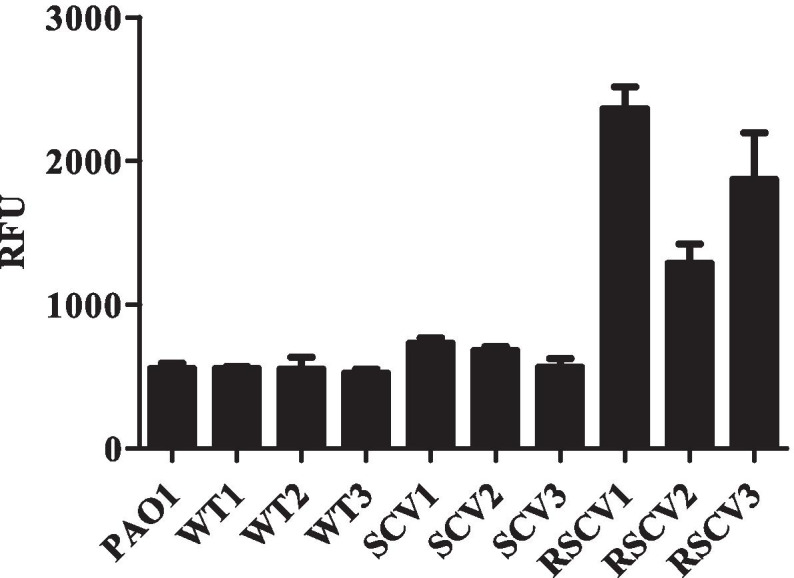
C-di-GMP levels of colonies that fail to disperse upon starvation measured using the *cdrA::gfp* reporter. Individual isolates were grown in M9 glucose medium overnight and 2 ml of culture was transferred to a 24 well plate in triplicate. GFP fluorescence and OD were measured using a Tecan plate reader. RFUs were calculated as GFP/OD_600_. Each data point is the average of three technical replicates

To further characterise the morphotypic variants isolated from starved biofilms, the isolates were tested for c-di-GMP production, biofilm formation and dispersal in response to starvation. All colonies with a WT phenotype had pCdrA::*gfp* expression levels (559 ± 11, 553 ± 80, 525 ± 25 RFU), that were similar to the parental strain (555 ± 42 RFU), indicating they have similar concentrations of c-di- GMP (Fig. 4). SCVs had slightly higher c-di-GMP levels (734 ± 37,682 ± 28,567 ± 58 RFU) compared to WT. All of the RSCVs had considerably higher c-di-GMP levels compared to WT, although RSCV2 (1289 ± 134 RFU) had only half of the c-di-GMP levels of RSCV1 (2367 ± 152 RFU) and RSCV3 (1871 ± 324 RFU). The RSCVs were also found to form pellicles at the air-liquid interface of planktonic cultures (data not shown), a phenotype characteristic of the high c-di- GMP mutant *wspF* [30].

**Fig. 5:**
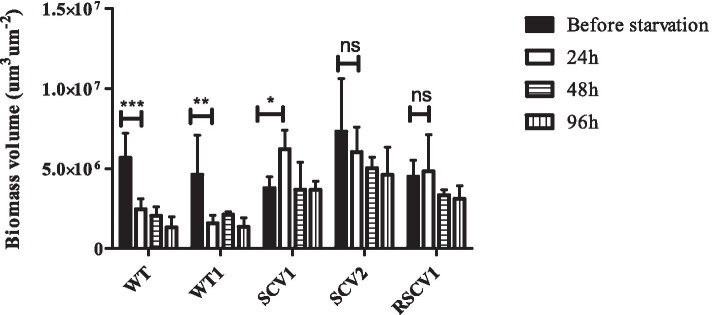
Starvation of variants. Biofilm starvation dispersal response of isolates collected after 4 d of biofilm starvation. Representative morphotypic isolates were grown in flow cells and starvation was induced at day 4. Biomass biovolumes were calculated as average of 3 independent experiments by CLSM images using IMARIS software. For each experiment 5 CLSM images were taken. Error bars represent standard deviations (*n* = 3). Asterisks indicate that the values are significantly different (Two-way ANOVA followed by Tukey’s post-test; *** < P 0.001, ** < P 0.01, * < P 0.05)

When these biofilm isolates were tested for their response to starvation induced dispersal, it was observed that the WT-like isolate, WT1, responded similarly to the parental WT strain (Fig. 5). WT1 showed 56.8% dispersal in the first 24 h post induction of starvation and showed no change in dispersal at 48 or 96 h. In contrast, none of the SCV or RSCV variants tested showed any significant dispersal upon starvation, even after 96 h.

### Complementation restores starvation induced dispersal phenotype to RSCV biofilm isolate

**Fig. 6:**
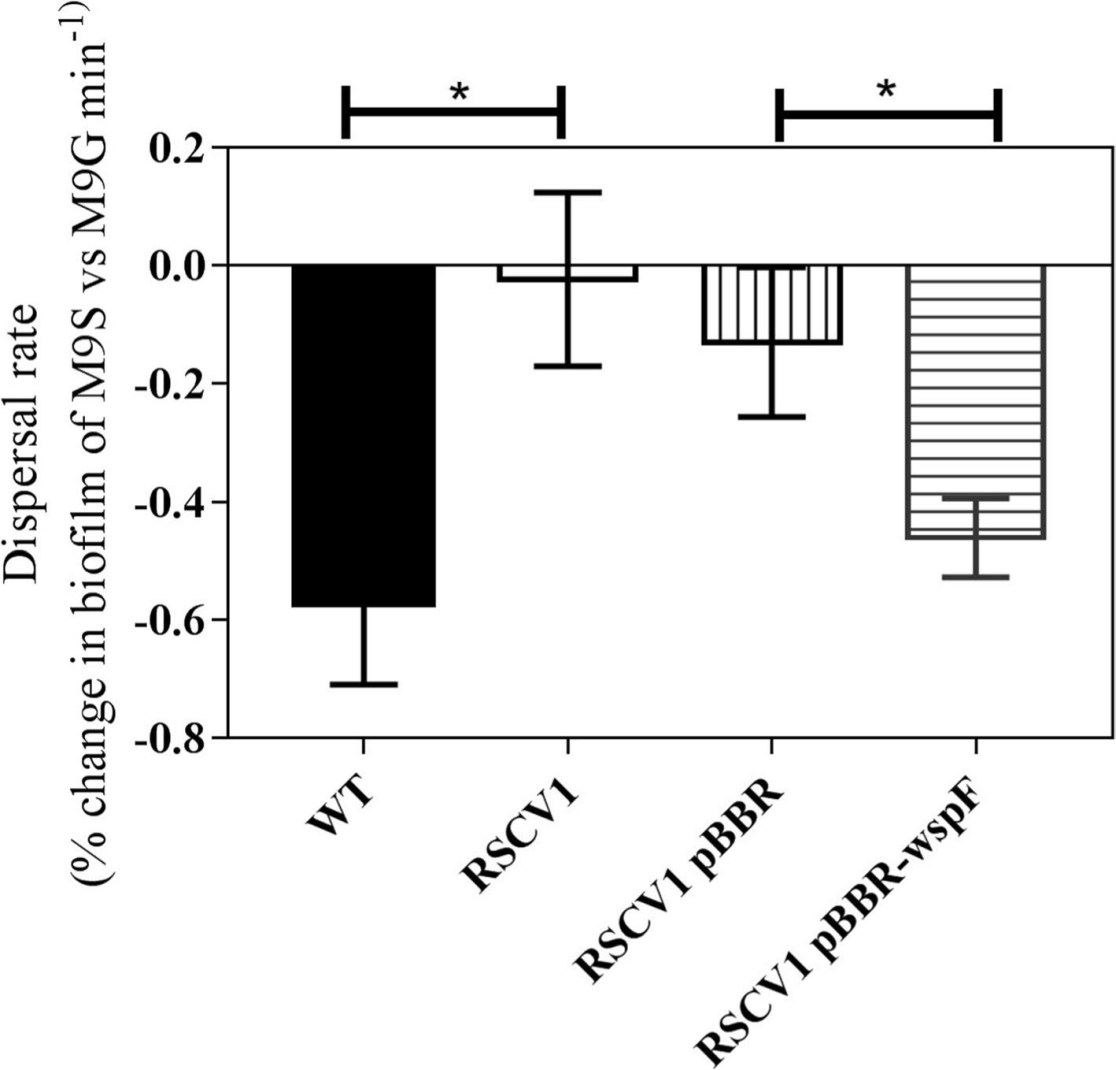
Complementation of non-dispersal phenotype of RSCV1 mutant under starvation conditions. Batch biofilms of WT, RSCV1, RSCV1/pBBR and RSCV1/pBBRwspF were cultivated for 6 h and starved. Biofilm biomass was quantified by crystal violet staining at different time points. Percentage biofilm changes were plotted against time and the rate of change of biofilm reduction was obtained through linear regression. Data shown are average values ± SD from 3 independent experiments. M9G-M9 medium containing glucose; M9S-M9 salts without carbon source. Asterisks indicate that the values are significantly different between strains (One-way ANOVA followed by Tukey’s post-test; * < P 0.05)

To confirm the effect of the mutations found in the SCV and RSCV isolates were responsible for their failure to disperse upon carbon starvation, we introduced wild-type copies of the genes affected. To carry out complementation analyses, RSCV1, WT5 and SCV5 mutants carrying plasmids with the respective wild type copies of *wspF*, *pilT* and *fha1* were generated. These three strains were selected because they had only a single gene that was mutated relative to the parental strain. All three colony morphotype variants, RSCV1, WT5 and SCV5 were restored to the wild type colony morphology upon complementation. Batch biofilms were cultivated with the mutants carrying either the empty vector (pBBR) or complementing clones (pBBR*wspF*, pBBR*pilT* or pBBR*fha1*), subjected to starvation conditions and dispersal was monitored. Analysis of dispersal phenotypes showed that only the RSCV1 mutant was complemented for this phenotype when WT *wspF* was provided *in trans* (Fig. 6). WT *P. aeruginosa* had a dispersal rate of 0.57 min^− 1^ whereas RSCV1 mutant and RSCV1/pBBR had dispersal rates of 0.02 min^− 1^ and 0.12 min^− 1^, respectively. The RSCV1 mutant carrying pBBR*wspF* was restored to almost WT levels of dispersion rates at 0.46 min^− 1^ suggesting that WspF is required for dispersal during starvation.

## Discussion

Many studies have shown that c-di-GMP plays a key role in regulating the switch between bacterial life styles, from free-living planktonic bacteria to sessile biofilms. Elevated levels of c-di-GMP are commonly associated with biofilm growth, and reductions in this second messenger can result in the dispersal of biofilms [34]. Dispersal cues responsible for altering the intracellular concentrations of c-di-GMP include oxygen limitation, cues such as NO and nutrient starvation [4, 9, 10, 15]. While starvation and NO can induce dispersal, it is frequently observed that dispersal is incomplete [3, 11]. It was recently demonstrated that the non-dispersing cells have increased levels of flavohemoglobin (Fhp) that scavenge and inactivate NO, thus preventing further dispersal [35]. Thus, it was of interest to investigate the effect of starvation as a dispersal cue in *P. aeruginosa* to assess the non-dispersing population.

Even though starvation did induce dispersal, 12.2% of the initial biomass remained in the flow cell after 3 d of continuous starvation (Fig. 1). Isolation and genetic characterisation of these dispersal insensitive colonies showed that they contained mutations. Since the WT morphotype colonies show dispersal similar to the parent strain, it is likely that these mutants would account for a smaller percentage of dispersal resistant mutants when compared to RSCV and SCV. The presence of morphological variants in the biofilm is a common observation [18, 24, 36] and morphotypic variants such as SCVs and RSCVs have been correlated with the persistence of *P. aeruginosa* during chronic infection of cystic fibrosis patients [33]. It is likely that these morphotypic variants arise in the population through natural mutational processes and that some of these natural mutants, in particular SCVs and RSCVs, were selected for in the dispersal resistant biomass. The isolates with WT morphotype harboured mutations in the *fliH* and *mexT* genes; however, their response to starvation and their intracellular c-di-GMP concentrations were similar to the parent strain. The *fliH* gene encodes a protein important for flagella biosynthesis [37] and a mutation would render *P. aeruginosa* nonmotile. The gene *mexT* encodes is a LysR-family regulator that modulates multi-drug efflux systems, resulting in increased resistance to diverse antibiotics [38]. Complementation of the mutants with *wspF* restored WT dispersal, while complementation with *pilT* or *fha1* did not. As for the WT isolates, none of the SCVs showed elevated levels of c-di-GMP. In contrast, all of the RSCVs contained *wspF* mutations and showed elevated levels of c-di-GMP, as shown using the pCdrA*::gfp* monitor (Fig. 6), although the concentration of c-di-GMP differed between isolates, possibly due to secondary mutations (e.g. *aguR*, PA4321, PA4543) [16]. Therefore, at least for these strains, the failure to disperse may be a consequence of being locked into the biofilm mode of growth. This is supported by the observation that the RSCV morphotypes do not appear in the biofilm effluent, but are strongly enriched in the non-dispersing biomass, even after 96 h of starvation.

These mutants most likely pre-exist in the biofilm and are strongly selected for under the conditions used here. It is likely that other triggers of biofilm dispersal, e.g. oxygen limitation or NO treatment may also select for high c-di-GMP producing mutants that are defective in dispersal. It would be of interest to determine if the same mutants arise that are impaired in dispersal, and would be the focus of future work which would include the specific signal response pathways for the different dispersal cues.

## Conclusions

The results presented here show that, as for other dispersal cues such as NO, some cells in the biofilm population do not respond to the dispersal cue. In the case of NO, the lack of response was associated with induction of the NO scavenging enzyme, Fhp. In contrast, while starvation also failed to disperse the entire biofilm, we found that this was at least in part, due to the presence of a population of genetic variants that over produce c-di-GMP and hence may lock the cells into a biofilm phenotype. Thus, repeated cycles of dispersal and regrowth may represent a strong selection pressure that favours mutants in the population that are unable to disperse, further reinforcing biofilm growth.

## Methods

### Bacterial strains and culture media

*P. aeruginosa* PAO1 WT and SCV or RSCV mutant derivatives generated in this study are listed in Table 1. *P. aeruginosa* PAO1 mini-*Tn7* CFP with a c-di-GMP reporter plasmid pCdrA*::gfp* (ASV) [39] was either cultured on Luria Bertani agar (LB broth, 10 g Tryptone, 10 g NaCL and 5 g yeast extract / L, with 1.5% bacto agar) or M9 minimal medium (48 mM Na_2_HPO_4_; 22 mM KH_2_PO_4_; 9 mM NaCl; 19 mM NH_4_Cl; 2 mM MgSO_4_; 0.1 mM CaCl_2_) supplemented with 20 mM glucose.

### Flow cell biofilms

Biofilms were cultivated in three-channel flow cells (channel dimensions, 1 × 4 × 40 mm) [40] and fed with M9 minimal medium supplemented with 20 mM glucose as a carbon source at a flow rate of 9 ml h^− 1^. Each channel was inoculated with 0.5 ml of diluted overnight culture containing approximately 1 × 10^8^ cfu ml^− 1^ [16].

### Confocal laser scanning microscopy and image analysis

Biofilm images were acquired using a Carl Zeiss Confocal Laser Scanning Microscope CLSM 780 (Carl Zeiss, Germany) with a 20x objective (LD plan- Neofluar 20x / 0.4 Korr M27). Two separate optical channels were used to image the biofilm biomass and c-di-GMP reporter fluorescence. A stably-expressed cyan fluorescent protein (CFP), inserted into the genome, was used to quantify the biomass and GFP expression used to quantify c-di-GMP [16]. Laser voltage was measured using a Sanwa™ CD800a digital multimeter and kept at 0.587 V throughout the experiment. Pinholes of 50 and 58 μm were used for CFP and GFP, respectively. An emission bandwidth of 440–503 nm was used for CFP and 497–598 nm was used for GFP. The master gain was set to 700 and the digital gain at 1.00 for acquisition of all of the images. Images were processed using ImageJ Version 1.47 [41].

### Starvation of planktonic and biofilm cultures

Starvation of planktonic bacterial cultures was achieved by transferring cultures grown overnight into sterile 50 ml Falcon tubes (Corning, Inc., U.S.) and centrifuging (10,000 X *g*) for 5 min at room temperature. Supernatants were discarded and bacterial cells were resuspended in M9 medium lacking a carbon source. The bacterial suspensions were then transferred to a new, sterile conical flask and incubated at room temperature with shaking (180 rpm). To initiate starvation in flow cell biofilms, a separate media bottle containing M9 medium without a carbon source was connected to the pump and used as feed for the biofilm [16].

### Determination of colony morphology

After 7 d of biofilm development, the flow cells were disconnected and bacterial cells were collected by flushing the biofilm several times with cold PBS using syringes connected to each end of the flow cell [42]. Biomass removal from the flow cells was validated by CLSM. The biomass was serially diluted with media (M9-glucose for normal growth, M9-without a carbon source for starvation) and plated onto LB agar plates. The plates were incubated for 3 d at room temperature and colonies were enumerated and morphology determined by use of a compound microscope (Zeiss Primo Star Compound Microscope) with 4x magnification.

### Variant analysis

Planktonic cultures of isolated colony variants were grown overnight and DNA from each sample was extracted using QIAamp DNA Mini Kit (Qiagen, Netherlands) according to the manufacturer’s protocol. The quantity of each DNA sample was determined using the Qubit DNA kit and the quality assessed using TapeStation. Libraries were prepared using the TruSeq DNA Sample Preparation Kit (Illumina, USA) and sequenced on the MiSeq (Illumina, USA) platform. The paired-end reads were trimmed to remove adapters and mapped to the *P. aeruginosa* reference genome (NCBI Reference Sequence: NC_002516.2) using CLC genomic workbench 6 (CLC bio, Denmark) [16].

### Complementation

Sequences coding for *wspF* (1008 bp), *pilT* (1035 bp) and *fha1* (1494 bp) were amplified from the *P. aeruginosa* wild type (WT) by PCR and cloned into the *Kpn*I-*Xba*I site of pBBRMCS-3. The resulting plasmids, pBBR*wspF*, pBBR*pilT* and pBBR*fha1* were introduced into RSCV1, WT5 and SCV5 respectively by electroporation. Colony morphology was assessed by compound microscopy (Zeiss) with 4X magnification. Batch biofilms were cultivated and dispersal assays carried out as follows; WT *P. aeruginosa* was grown overnight in LB supplemented with 50 μg ml^− 1^ of tetracycline. Subsequently, overnight cultures were washed in M9 medium and resuspended to OD_600_ = 1. Twenty μl of the suspension was added to 1 ml of fresh M9 medium supplemented with 0.4% w/v glucose in each well of a 24 well plate to a final OD_600_ ~ 0.02. The 24 well plates were incubated for 6 h at 37 °C with 180 rpm shaking. At 6 h, the medium was replaced with fresh M9 salts with or without 0.4% w/v glucose. To quantify biofilms, the medium was aspirated and each well washed once before staining with 0.1% crystal violet for 20 min, at which time the crystal violet solution was removed and each well washed twice with 1 X PBS. The remaining crystal violet was dissolved in ethanol and quantified using a microtitre plate reader (Tecan infinite pro M200). Biofilm biomass changes due to starvation at each time point was compared to the control grown in M9 glucose and calculated as (OD_550_ starved – OD_550_ control)/OD_550_ control × 100%. The percentage biofilm changes were plotted against time and the rate of change of biofilm reduction was obtained by determining the slope of the line. Data were obtained from three independent replicates with two technical replicates each. ANOVA analysis was carried out using GraphPad Prism v8.1.0 followed by Tukey’s post-test.

## Data Availability

All data generated or analyzed during this study are included in this published article. The genome sequence data supporting the conclusions of this article are available in the NCBI SRA database with BioProject accession number PRJNA733671 (http://www.ncbi.nlm.nih.gov/bioproject/733671).

## Abbreviations

RSCV: rugose small colony variants
SCV: small colony variants
WT: wild type
c-di-GMP: cyclic diguanylate
SNP: single nucleotide polymorphism
SNV: single nucleotide variant
